# Clinical Practice Guidelines on the Treatment of Patients with Cleft Lip, Alveolus, and Palate: An Executive Summary

**DOI:** 10.3390/jcm10214813

**Published:** 2021-10-20

**Authors:** Aebele B. Mink van der Molen, Johanna M. M. van Breugel, Nard G. Janssen, Ronald J. C. Admiraal, Leon N. A. van Adrichem, Frank Bierenbroodspot, Dirk Bittermann, Marie-José H. van den Boogaard, Pieter H. Broos, Janet J. M. Dijkstra-Putkamer, Martine C. M. van Gemert-Schriks, Andrea L. J. Kortlever, Chantal M. Mouës-Vink, Henriette F. N. Swanenburg de Veye, Nanouk van Tol-Verbeek, Christl Vermeij-Keers, Hester de Wilde, Anne Marie Kuijpers-Jagtman

**Affiliations:** 1 Department of Plastic and Reconstructive Surgery, University Medical Center Utrecht, 3584 CX Utrecht, The Netherlands; J.M.M.vanBreugel-4@umcutrecht.nl (J.M.M.v.B.); leon@vanadrichem.com (L.N.A.v.A.); 2 Department Maxillo Facial Surgery and Dentistry, University Medical Center Utrecht, 3584 CX Utrecht, The Netherlands; n.g.janssen-2@umcutrecht.nl (N.G.J.); dirk@bittermann.nl (D.B.); 3 Department of Oto-Rhino-Laryngology, Radboud University Medical Center, 6500 HB Nijmegen, The Netherlands; rjc.admiraal@planet.nl; 4 Department Maxillo Facial Surgery, Isala, 8025 AB Zwolle, The Netherlands; f.bierenbroodspot@isala.nl; 5 Department of Clinical Genetics, University Medical Center Utrecht, 3584 CX Utrecht, The Netherlands; m.j.h.vandenboogaard@umcutrecht.nl; 6 Knowledge Institute of the Federation of Medical Specialists, 3528 BL Utrecht, The Netherlands; p.broos@jbz.nl (P.H.B.); a.kortlever@kennisinstituut.nl (A.L.J.K.); 7 Department of Speech Therapy, Medical Center Leeuwarden, 8934 AD Leeuwarden, The Netherlands; j.dijkstra-putkamer@antonius-sneek.nl; 8 Academic Dentistry Center (ACTA), 1081 LA Amsterdam, The Netherlands; m.schriks@acta.nl; 9 Department of Plastic and Reconstructive Surgery, Medical Center Leeuwarden, 8934 AD Leeuwarden, The Netherlands; Chantal.moues@znb.nl; 10 Department Medical Psychology, University Medical Center Utrecht, 3584 CX Utrecht, The Netherlands; h.deveye@umcutrecht.nl; 11 Association of Educationalists in the Netherlands, 3527 GV Utrecht, The Netherlands; nanoekvantol@gmail.com; 12 Dutch Association for Cleft Palate and Craniofacial Anomalies, 3643 AE Mijdrecht, The Netherlands; cvermeijkeers@hotmail.com; 13 Department of Speech Therapy, University Medical Center Utrecht, 3584 CX Utrecht, The Netherlands; h.dewilde@umcutrecht.nl; 14 Department of Orthodontics, University Medical Center Groningen, University of Groningen, 9713 GZ Groningen, The Netherlands; a.m.kuijpers-jagtman@umcg.nl; 15 Department of Orthodontics and Dentofacial Orthopedics, School of Dental Medicine, Medical Faculty, University of Bern, CH-3010 Bern, Switzerland; 16 Faculty of Dentistry, Universitas Indonesia, Jakarta 10430, Indonesia

**Keywords:** cleft lip, alveolus and palate, clinical practice guideline, quality of health care, recommendation clinical care, treatment

## Abstract

Significant treatment variation exists in the Netherlands between teams treating patients with cleft lip, alveolus, and/or palate, resulting in a confusing and undesirable situation for patients, parents, and practitioners. Therefore, to optimize cleft care, clinical practice guidelines (CPGs) were developed. The aim of this report is to describe CPG development, share the main recommendations, and indicate knowledge gaps regarding cleft care. Together with patients and parents, a multidisciplinary working group of representatives from all relevant disciplines assisted by two experienced epidemiologists identified the topics to be addressed in the CPGs. Searching the Medline, Embase, and Cochrane Library databases identified 5157 articles, 60 of which remained after applying inclusion and exclusion criteria. We rated the quality of the evidence from moderate to very low. The working group formulated 71 recommendations regarding genetic testing, feeding, lip and palate closure, hearing, hypernasality, bone grafting, orthodontics, psychosocial guidance, dentistry, osteotomy versus distraction, and rhinoplasty. The final CPGs were obtained after review by all stakeholders and allow cleft teams to base their treatment on current knowledge. With high-quality evidence lacking, the need for additional high-quality studies has become apparent.

## 1. Introduction

Among the most common congenital abnormalities are clefts of the lip, alveolus, and palate. In Western Europe, the prevalence is approximately 1.7 per 1000 live births [1]. The treatment of orofacial clefts in children is usually carried out by a multidisciplinary team of specialists in consultation with the patient and their parents. Teams offer individualized care from the antenatal period or from birth until treatment, including possible orthognathic surgery and secondary corrections has been finished at approximately 22 years of age.

In the Netherlands, significant practice variation exists between cleft teams, and parents, patients, and healthcare professionals consider this to be confusing and undesirable. This became clear in 2009 during the process of developing clinical practice guidelines (CPGs) on counseling after detecting clefts prenatally [2,3]. To ensure best practices and decrease practice variation and confusion, it is important that professionals determine the scientific foundation of medical interventions and define the standard of care that can be expected by both patients and parents. Therefore, it was judged to be beneficial to create evidence-based CPGs in order to realize more standardized postnatal cleft care throughout the Netherlands. Potentially, these results could also be applied abroad.

The general aim of the guidelines is to optimize care for patients with cleft lip, alveolus, and/or palate based on sound scientific evidence where possible. In addition, compiling the guidelines based on current evidence would reveal existing knowledge gaps. This could steer future research, which may provide reliable and independent information about treatment methods that are currently lacking.

The objective of this report is to describe the development of the CPGs and to share the main recommendations, knowledge, and experience we obtained while writing them. Moreover, we will indicate gaps in current knowledge regarding cleft care that became apparent during the process. The full text of the guidelines has been translated into English and can be found at: https://ern-cranio.eu/resources/clinical-guidelines/ (accessed 19 October 2021), and the original text in Dutch is available at: https://richtlijnendatabase.nl/richtlijn/behandeling_van_patienten_met_een_schisis/startpagina_schisis.html (accessed 17 October 2021).

## 2. Materials and Methods

**Figure 1:**
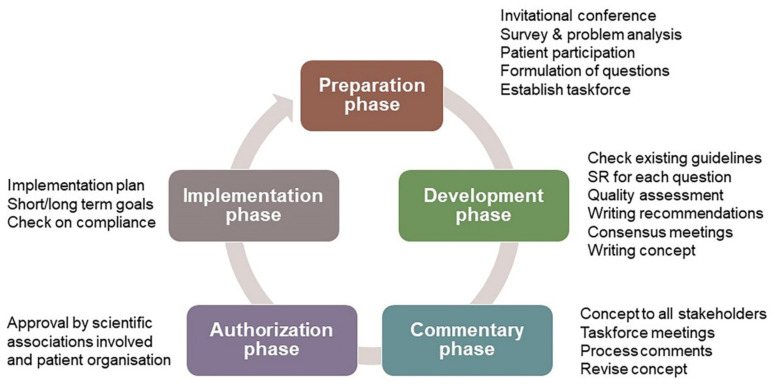
Overview of the phases of CPG development.

This CPG was drafted according to the standard for guideline development by the Guidelines Advisory Committee of the Dutch Association of Medical Specialists’ Quality Council (“Richtlijnen 2.0”) and the Appraisal of Guidelines for Research and Evaluation II (AGREE II) instrument (www.agreetrust.org (17 September 2021)). The AGREE II is widely accepted for assessing the quality of guidelines. Professional support was given by two experienced epidemiologists from the Knowledge Institute of the Federation of Medical Specialists in the Netherlands (Kennisinstituut, www.kennisinstituut.nl (17 September 2021)). Their support ensured a systematic and consistent approach during guideline development (Figure 1). During the preparation phase in 2013, an invitational conference was organized for all stakeholders, including patients, to define the areas of uncertainty in cleft care. The CPGs were developed between 2013 and 2016 with additional modules developed and added from 2017 to 2019 as supplementary funding became available. A detailed description of the methods can be found in Supplementary Materials S1.

## 3. Results

### 3.1. Database Search

**Figure 2:**
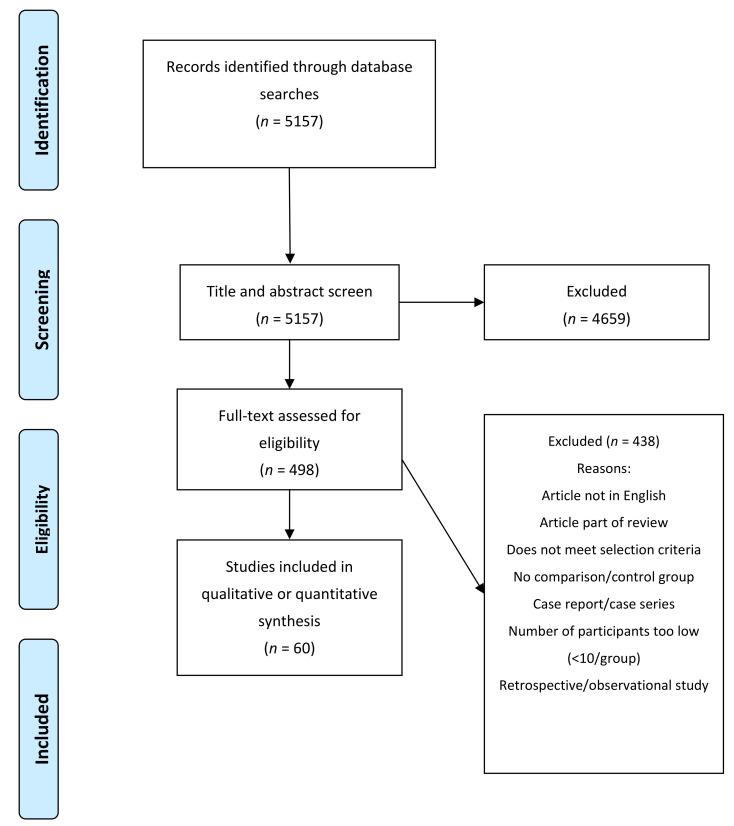
PRISMA flowchart of our search, screening, and inclusion strategy.

The initial literature search identified 5157 articles, which were then screened based on their titles and abstracts; 4659 articles were subsequently excluded, including duplicates. The full texts were obtained for the remaining 498 articles when possible. Another 438 articles were excluded for reasons shown in Figure 2. Finally, 60 studies were eligible to base the development of the guidelines upon. These 60 studies, that were used for the conclusions, are listed in Supplementary Materials S2. No systematic searches were performed for the chapters of genetic testing and dentistry.

### 3.2. Clinical Questions and Recommendations

Readers of this executive summary are advised to consult the full text of the CPG for further information. 

#### 3.2.1. Genetic Testing

Clinical question: When should children with a cleft lip, alveolus, and/or palate undergo genetic testing? 

A systematic search was performed, but the search did not yield any article answering the question. We also found no comparative studies about the effectiveness of genetic testing for cleft lip and palate. Therefore, the guideline working group decided on a consensus method. 

Recommendations: All patients with an orofacial cleft should be referred to a specialized (tertiary) center for clinical genetics, preferably before their first operation. A clinical geneticist should be involved in performing case genetic testing. When growth and feeding problems are present or developmental delay with associated abnormalities, and a syndrome diagnosis or chromosomal abnormality is suspected, immediate referral is recommended. For an isolated cleft palate, prior to the first operation, a single nucleotide polymorphism (SNP) array is recommended, and then additional genetic testing can be considered, such as whole-exome sequencing (WES) or a gene panel. For cleft lip, alveolus, and palate, or a child with cleft lip with or without cleft alveolus, additional genetic testing must be considered and the (dis)advantages discussed thoroughly with the parents [3,4,5,6,7,8,9,10,11]. Since the writing of this chapter, some relevant papers on WES for cleft lip and palate have been published, but these do not change the recommendation [12,13,14]. 

#### 3.2.2. Administering Food

Clinical question: What is the best manner in which to feed children with cleft lip, alveolus, and/or palate immediately after birth and immediately after surgery?

Recommendations: The goal is to have a comfortable feeding process for both the parent and the child. Start oral feeding as soon as possible postnatally and postoperatively. Tube feeding is not advised for use as the only feeding method. A knowledgeable and experienced speech therapist should be a member of each cleft team to ensure development of a normal drinking and eating pattern taking into account the effect of the deformity on these processes. 

Alongside the parents, decide on an individualized method of administering food to their infant. Consider various feeding methods, including breast and bottles, as well as feeding positions. Take the parents’ capabilities into account during the prenatal preparation phase and when offering support for potential problems with feeding [15,16,17,18,19,20,21,22].

#### 3.2.3. Lip and Palate Surgical Repair

##### Timing of Repair

Clinical question: What factors are involved in determining the timing for surgical repair of a child’s cleft lip and palate?

Recommendations: Surgical lip repair should be performed in the first 6 months after birth. The cleft team should determine their preferred approach to support the parents in making a decision, but also discuss different approaches with the parents. The soft palate should be closed during the first year of the infant’s life, with the hard palate repaired later if aiming for optimal maxillary growth. However, the hard and soft palate should both be closed during the first year of life if optimal speech development is pursued. The Eurocleft checklist should be completed prior to surgery and intra-oral photographs taken during surgery as needed. Any abnormalities should be documented in the surgical report [23,24,25,26,27,28,29,30,31,32,33,34,35].

##### Repair Technique

Clinical question: Is there a preferred surgical technique for closing Veau class 1 and 2 type soft palate in children who have a cleft palate (with or without other defects)?

Recommendations: Move the palatal musculature to a more anatomically correct position during palate repair (connecting the levator muscles in the midline and more posterior), such as transposition of the muscles with reconstruction of the levator sling using a Furlow or Von Langenbeck technique, to achieve better results for speech. For a wide cleft palate, do not use a Furlow double opposing Z-plasty, as it has an increased risk of fistula formation. In addition, it is preferable to not use the Wardill–Kilner pushback technique. To improve approximation of the wound margins, use everting techniques (sutures). To minimize the complication risk, the surgeon should use the (combination of) surgical technique(s) for palate repair in which he/she is most experienced. 

For lip repair, the surgeons should also use the (combination of) technique(s) that they are most experienced in to ensure an optimal functional and aesthetic result while minimizing the risk of complications [34,36,37,38,39,40,41].

#### 3.2.4. Hearing Problems

Clinical question: What factors are involved in treating hearing loss in children with cleft lip, alveolus, and/or palate?

Recommendations: Check the neonatal hearing screen results for each child with these deformities and perform periodic audiology check-ups up to 3 to 4 years of age, followed by check-ups by the ear, nose, and throat (ENT) specialist if indicated. It is recommended that these examinations align with the surgical protocol [42,43].

Check hearing at least once after the insertion of grommets to rule out perceptive hearing loss. Grommets should be inserted in children with cleft palate or cleft lip, alveolus, and palate only if indicated (see CPG on Otitis Media in children [44]). In addition, the audiological findings and speech-language results should be taken into consideration. 

Hearing aids can be an alternative to grommets, as they have comparable audiological results but with fewer complications and long-term negative sequelae [45,46]. However, in the long-term, grommets are the most cost-effective approach, followed by hearing aids. A wait-and-see approach is the least cost-effective [47].

#### 3.2.5. Hypernasality

##### Diagnosis

Clinical question: What is the recommended strategy for diagnosing velopharyngeal dysfunction (VPD) in children with cleft lip, alveolus, and/or palate? 

Recommendations: No eligible studies were found. Consequently, the following recommendations were based on a consensus of the working group. 

The diagnosis of VPD should be multi-disciplinary, with input from at least a cleft surgeon, speech therapist, and ENT specialist. After primary palate repair, VPD should be diagnosed only when 6 months of specialized speech therapy has not had adequate results, provided that a sufficiently long and mobile soft palate is found upon intra-oral inspection and the patient is able to adequately follow instructions. Diagnostic imaging should be as complete as possible and include oral inspection, mirror tests, acoustic nasometry, and nasal endoscopy to confirm VPD. Nasal endoscopy is only indicated if the child is likely to cooperate (usually from the age of 3.5 years). Use videofluoroscopy as an alternative. It is preferred that the speech therapist be present during nasal endoscopy and videofluoroscopy. The nasal endoscopy should be recorded as photos or video. One year after speech-enhancing surgery, the preoperative examinations should be repeated to assess the effect, except possibly the nasendoscopy/videofluoroscopy. Repeat the nasal endoscopy/videofluoroscopy after speech-enhancing surgery if the examinations performed after 6 months of specialized speech therapy indicate (remaining) VPD with insufficient intelligibility. Dynamic MRI is not recommended as a routine procedure within the diagnostic process [48,49,50,51,52,53,54].

##### Surgical Treatment

Clinical question: What surgical treatment is recommended for VPD in children with a cleft lip, alveolus, and/or palate?

Recommendations: The specific surgical technique should be chosen based on the results of the preoperative speech assessment and other examinations, such as nasal endoscopy or videofluoroscopy. Before performing pharyngoplasty, consider intravelar palatoplasty with repositioning of the palatal muscles if the patient has persistent VPD despite a previously closed palate. For persistent VPD despite repositioning the palatal muscles, pharyngoplasty can be considered based on the results of repeated diagnostic tests, such as nasal endoscopy or videofluoroscopy. Simple palatoplasty is preferred for a submucosal cleft palate, rather than combined palatoplasty and pharyngoplasty. Only use fat injection (lipofilling) in a research context [55,56].

#### 3.2.6. Bone Grafting Procedures

##### Timing of Bone Grafts

Clinical question: What factors are involved in determining the timing for bone grafts in patients with cleft lip, alveolus, and/or palate (unilateral or bilateral)?

Recommendations: Closing the alveolar cleft with an early secondary bone graft is preferable. Base the timing on the position and root formation stage (½–⅔) of the maxillary canine on the cleft side. The timing can be moved forward by the presence and eruption of a lateral incisor on the cleft side. Decide on the timing based on consultations with the orthodontist. A tertiary bone grafting procedure should be considered only for children who have not undergone a (secondary) bone graft or if insufficient bone is available in the former alveolar cleft area for later work, such as a dental implant. The tertiary bone grafting procedure can be approached during adulthood [57,58,59,60,61,62,63,64].

##### Bone Graft Technique

Clinical question: Is there a preferred bone grafting material for alveolar cleft reconstruction?

Recommendations: The alveolar cleft can be reconstructed using bone from the iliac crest if a large volume is required, or from the chin supplemented by bone substitute. There is not sufficient evidence from the literature to make a recommendation for a certain bone substitute. Bone substitute without autologous bone should be used only in a research context [58,65,66,67,68].

#### 3.2.7. Orthodontic Treatment

##### Nasoalveolar Molding (NAM)

Clinical question: Is NAM indicated for a complete unilateral or bilateral cleft lip, alveolus, and/or palate?

Recommendation: Be careful with the application of NAM in this context and use NAM only when preparing for or performing a clinical trial [69,70].

##### Maxillary Protraction

Clinical question: Is maxillary protraction appropriate for use in children with a cleft lip, alveolus, and/or palate?

Recommendations: In general, maxillary protraction should not be performed using a facemask and dentally anchored orthodontic device in growing children with a cleft lip, alveolus, and/or palate and deficient growth of the maxilla. This technique can be considered when a slight midfacial deficiency is present and (later) orthognathic surgical treatment is not expected, or if the patient has certain favorable facial characteristics (see the full guidelines). If maxillary protraction is to be applied using a dentally anchored orthodontic device and facemask, inform the patient and/or parents of the limitations of the procedure and that whether an osteotomy of the maxilla will be needed can only be judged at the end of the growth period [71,72].

##### Orthodontic Retention

Clinical question: What type of orthodontic retention is most effective in children with a cleft lip, alveolus, and/or palate for stabilizing the tooth position and maxillary dental arch shape over the long term?

Recommendations: Use the same type of retention to retain the anterior tooth position as for a patient without a cleft. In addition, use a removable orthodontic retainer to maintain the transverse dimensions of the maxillary dental arch. Such a retainer should be worn at night for life and be checked at least once every two years [73].

#### 3.2.8. Psychosocial Guidance

Clinical question: Is psychosocial support effective as part of the multidisciplinary treatment of children with cleft lip, alveolus, and/or palate?

Recommendations: Screen both the patient and their parents for psychosocial problems after birth and when the child is 2–3 years old, 5 years old, 10–11 years old, and 17 years old. The screening at these contacts should include patient-related factors, such as possible learning problems, well-being, fear of medical procedures, and acceptance problems, as well as family and parent-related factors (i.e., grief, parenting style, and acceptance problems). 

The same validated instrument should be used by the cleft teams for screening, such as the Strength and Difficulties Questionnaire (SDQ) and Family Questionnaires, in addition to the regular conversations with parents and/or patients during visits [74,75]. The parents and patient should be offered additional diagnostic tests or treatments based on the results of the screening. The cleft team should include a behavioral expert with post-academic training, such as a clinical psychologist or special education generalist, as well as a social worker to assist in the diagnosis and treatment of complex psychosocial problems [76,77].

#### 3.2.9. Dentistry

Clinical question: What role do dentists have on the cleft team and what role do general practitioners play in the dental care of children with a cleft lip, alveolus, and/or palate?

The pathogenesis of dental caries and periodontal disease does not differ between individuals with and without orofacial clefts. However, in children with clefts, the prevalence of caries is higher in both the deciduous and permanent dentition due to anatomical limitations of the oral cavity and teeth, bad oral hygiene, prolonged duration of oral appliance use, lower socioeconomic status of the families, and over-indulging parents and caretakers [78,79,80]. Therefore, it is important to guarantee access to dental care for all children with clefts. This is a background question on the organization of care, which is difficult to translate into a search question for a systematic review. A recently published systematic review of CPGs for oral health care in children with clefts identified seven, but none of them were rated as high quality for “Rigor of Development” [81]. This shows that there is no high-quality evidence on which to base a recommendation for organization of care. Therefore, the working group opted for a consensus method.

Recommendation: A dentist with an interest in pediatric dentistry should be a member of the cleft team. They should see the child as soon as their first deciduous teeth have erupted (at the age of 6 to 12 months). Then, they should check the child’s dentition at 5 years old during a team consultation. This should be in addition to periodic monitoring by a general dentist, who must be informed of the treatment implemented by the cleft team. The question of who provides the general oral health care for the child should be discussed [82,83]. The general dentist should check the dentition of a child with clefts at least every 6 months and should contact the cleft team and arrange for consultation if necessary.

#### 3.2.10. Osteotomy versus Distraction Osteogenesis

Clinical question: What are the factors involved in deciding on forward displacement of the maxilla via Le Fort I osteotomy vs. distraction osteogenesis in patients with a cleft lip, alveolus, and/or palate?

Recommendations: A Le Fort 1 osteotomy, alone or in combination with a setback osteotomy of the mandible, should be considered in patients with a sagittal jaw discrepancy that cannot be resolved by orthodontic treatment alone due to its size. Consider distraction osteogenesis only for large sagittal discrepancies. Both Le Fort I osteotomy and maxillary distraction have specific benefits and drawbacks that may aid in decision-making [84,85].

#### 3.2.11. Rhinoplasty

Clinical question: What are the indications for a rhinoplasty procedure in children with cleft lip (with or without cleft alveolus and palate)?

Recommendations: Primary correction of the nostril, as well as the position of the caudal septum and columella of the nose, should be considered during primary lip repair. To limit the total number of surgical procedures, it is preferable to delay secondary nasal surgeries until mid-face growth has ceased and any planned orthognathic surgery is completed [74,75].

## 4. Discussion

### 4.1. Guideline Principles

The primary aim of CPGs is to support healthcare providers in their practice. In addition, CPGs are a valuable source of information to quickly update healthcare providers’ knowledge on state-of-the-art treatments. Ideally, CPGs are based on evidence and, in the absence of evidence, the methodology for developing guidelines allows for the inclusion of expert opinions, as done in the present CPGs. Consequently, the resulting recommendations often have the lowest level of evidence. Guidelines are not legal instruments, but they include as many insights and recommendations based on evidence as possible. Healthcare professionals should follow the guidelines whenever possible to provide good quality care.

Although the findings presented in these CPGs are based on systematic reviews of the international literature, the expert opinions are mainly a result of consensus meetings among Dutch experts. As the Netherlands are relatively homogeneous in terms of socio-economic status, education, health, nutrition, and access to cleft care compared to some other countries, foreign caregivers may have to take local factors into account to ensure optimal care. For example, the sequence of lip and palate repair may be changed to ensure parents bring in their children for the second procedure instead of opting solely for lip repair that satisfies their basic needs aesthetically.

These guidelines were written for the treatment of patients with so-called “isolated” or non-syndromic clefts of the lip, alveolus, and palate. However, many of the recommendations may also apply to patients with an orofacial cleft in combination with other anomalies, though the approaches may need to be adjusted for the underlying disease or situation, such as the use of certain drugs. Moreover, these recommendations are based primarily on general evidence regarding the optimum care of an average patient, and care providers can deviate from the guidelines for individual cases, if necessary, based on their professional autonomy.

Obviously, there are many other relevant issues that could have been addressed. However, it was impossible to cover all topics due to time constraints and the availability of funding. Prioritizing was inevitable, for which the patients and parents we consulted played an important role. They were especially confused by the variation in treatment protocols between different cleft teams in our country, with all teams claiming that their protocol was of a high standard. Their input guided the selection of questions addressed in our guidelines, and the working group is responsible for the final decision on the most relevant issues. While revising and updating the guidelines in the future, as is routine for Dutch CPGs, new subjects may be added. 

### 4.2. Findings

Overall, the lack of scientific evidence in the field of cleft lip, alveolus, and palate was striking. High-quality studies, such as relevant randomized controlled trials, good cohort studies, and systematic reviews, remain scarce. An overview of the main gaps in knowledge is provided in Supplementary Materials S3. As a clinician, it can be disappointing to see that writing up years of clinical experience does not translate into measurable evidence unless it is part of a well-designed study. Here, cleft teams still have work to do. Randomized controlled trials and long-term outcome studies are difficult to organize and require, in addition to funding, a lot of organization, cooperation, and lasting discipline. Good design, large sample sizes, and clear subphenotyping are needed to obtain sufficient statistical power, making a multicenter approach almost inevitable [86].

The surgeon’s skills, including education, personal training, experience, workload, and working environment, are likely an important factor in the outcome, but unfortunately remain a hard to measure variable in surgery-related studies, and the place of robotic cleft surgery needs to be defined [87]. Furthermore, a uniform and validated method to assess, quantify, and document VPD is not available. Moreover, reliable, reproducible assessment of speech remains a challenging field. Comparable speech assessment requires both validated tools and systematic consensus training, which is not always available. Therefore, calibration is a key element in future studies and trials, including the surgical portion of any multicenter study. Other factors influencing the quality and success of cleft care are the family and society in which a child grows up. Being raised by a single parent, having parents with a challenged employment status, having harmony among siblings, getting accepted in school, having friends and being able to play with them, and negotiating day to day interactions with peers, elders, and juniors all have their effect on the psychology and social rehabilitation of these children and, thus, on cleft care.

Finally, there is the problem of inadequate outcome measures. What defines good outcomes for cleft care? Should it be defined from the doctor’s perspective or the patient’s? Should outcome measures focus on aesthetics, function, or quality of life as a whole? At what expense and burden of care? The introduction of patient-related outcome measures will give us at least some of the answers. Future studies would benefit from international consensus on how to measure outcomes in cleft care. The development of the ICHOM standard set for cleft lip and palate is a step forward (www.ichom.org), but more work needs to be done.

Because of the limited quality of the evidence from the available literature, some of the recommendations in this CPG are rather general and consensus statements were needed. The working group weighed and formulated these statements as carefully as possible, leaving room for individualized solutions based on the patient. Readers may not agree with some of the conclusions this working group formulated when evidence levels were low. Nevertheless, publication of this CPG is, in our opinion, important because it forms a basis upon which others can build and will hopefully prevent redundancy in future research. It clearly shows where we stand and what needs to be done to improve our results: design and perform studies of high quality based on unambiguous subphenotyping of the cleft lip, alveolus, and palate. This CPG is a ‘living document’ divided into separate modules that enable certain topics to be updated as soon as new scientific evidence becomes available.

## 5. Patents

No patents resulted from the work reported in this manuscript.

## Data Availability

All data resulting from our extensive literature search can be found in the full-text of the clinical practice guidelines at https://ern-cranio.eu/resources/clinical-guidelines/ (accessed on 19 October 2021).

## Supplementary Materials

The following are available online at https://www.mdpi.com/article/10.3390/jcm10214813/s1, Supplementary Materials S1: Methods, Supplementary Materials S2: List of 60 references used for the conclusions of this guideline, and Supplementary Materials S3: Current gaps in knowledge.
