# Supplementary material for: Clinical Practice Guidelines on the Treatment of Patients with Cleft Lip, Alveolus, and Palate: An Executive Summary
Source: J Clin Med. 2021 Oct 20;10(21):4813. doi: 10.3390/jcm10214813 (PMC8584510; doi:10.3390/jcm10214813)
Supplement: Supplementary file 1 [file jcm-10-04813-s001.zip › jcm-1332346-supplementary.pdf]

Supplementary Material S1

Methods

1. Initiative and Task Force

The Dutch Society for Plastic and Reconstructive Surgery (NVPC) initiated the development of this CPG. A multi-disciplinary guideline working group consisting of representatives from all relevant specialties involved in cleft care and from the Dutch Association for Cleft Palate and Craniofacial Anomalies (NVSCA) was established in 2013. Input from (former) patients was guaranteed through a focus group. In addition, the patient and parent organization Dutch Association for People with Disabilities and their Parents (BOSK) was allowed to assess the drafted guidelines and make suggestions to improve them during the commentary phase.

2. Guideline development

The CPG was drafted according to the requirements of the Guidelines Advisory Committee of the Dutch Association of Medical Specialists' Quality Council ("Richtlijnen 2.0") and the Appraisal of Guidelines for Research and Evaluation II (AGREE II; www.agreetrust.org) instrument, an internationally accepted tool for assessing guideline quality. Professional support was given by the Knowledge Institute of the Federation of Medical Specialists in the Netherlands (Kennisinstituut) in the form of two experienced epidemiologists. Their support ensured a systematic and consistent approach during the guideline development. During the preparation phase in 2013, an invitational conference was organized for all stakeholders, including patients, to define the areas of uncertainty in cleft care. The CPG was developed between 2013 and 2016 with additional modules developed and added from 2017 to 2019 as supplementary funding became available.

3. Intended users of the guidelines

The CPGs are intended for healthcare professionals involved in cleft care: general practitioners, midwives, obstetricians, pediatricians, ENT specialists, plastic surgeons, orthodontists, maxillofacial surgeons, clinical geneticists, specialized nurses, (pediatric) dentists, speech therapists, remedial educationalists, medical psychologists, and social workers. The secondary target group is patients, their parents, and those who surround them, though the guidelines are not written using laymen's terminology.

4. Delineation of the guidelines

A broad range of treatments and interventions related to cleft care represent areas of uncertainty and are potentially subjects for CPGs. The preparatory phase started by defining which areas of uncertainty had priority and could be translated into clinical questions. The working group was forced to prioritize and select topics to meet the available financial resources. Patient and treating physician's wishes, the relevance of the problems for children and parents, and rapid scientific developments that need to be acted upon were taken into consideration when selecting the topics for this CPG.

The guidelines focus primarily on the treatment of patients aged 0 to 22 years with isolated clefts of the lip, alveolus, and palate. The guidelines also apply to subphenotypes of these deformities: cleft

lip with or without cleft alveolus; cleft lip, alveolus, and palate; and cleft palate only (without other anomalies).

5. Clinical questions and outcome measures

The chairperson and advisor (epidemiologist) drafted clinical questions based on the invitational conference and discussions with the aforementioned parties. The members of the working group elaborated and confirmed these questions, and outcome measures relevant to the patient were determined for each. The working group ranked these outcome measures according to their relative significance as critical, important, or unimportant. Clinically relevant differences for a certain outcome measure were defined where possible, such as when an outcome's improvement translates into an improvement for the patient.

6. Literature search, data extraction, risk of bias, and quality of evidence assessment

First, a medical information specialist searched for existing foreign CPGs and systematic reviews in Medline (OVID), Embase, and the Cochrane Library between 2000 and March 12, 2014, and between 2000 and August 2017 for additional modules on naso-alveolar molding and Le Fort osteotomy. The literature searches aimed to identify systematic reviews, randomized controlled trials (RCTs), controlled clinical trials (CCTs), and observational studies published in Dutch or English.

Specific search terms from each of the clinical questions were then used to search for publications in the same electronic databases. The initial search focused on studies with the highest level of evidence. The working group members selected useful articles based on pre-determined inclusion and exclusion criteria. The inclusion criteria were having been written in English or Dutch, sample size $\geqslant$ 10 per group, and a prospective study design. The exclusion criteria were no control or comparison group, case report, or case series. The search strategy is described in more detail in the full guidelines. A manual search based on the references of the included articles was also performed. One observer extracted the data on the type of study, patient characteristics, comparisons, interventions, outcome measures, follow-up period, and effect size. A second observer checked the extracted data.

Risk of bias was assessed for all included RCTs and non-randomized observational studies. The Grading of Recommendations, Assessment, Development and Evaluations (GRADE) approach was used to grade the quality of evidence and make recommendations [1]. GRADE was used to rate the body of evidence at the outcome level. The working group discussed the evidence during several meetings and drew up considerations, finally leading to recommendations for each clinical question. The most important recommendations derived through the CPG development process are described in this report.

7. Commentary and authorization phase

Expert meetings to discuss the findings, knowledge gaps, and expert opinions led to a first draft of the guidelines per topic. These drafts were made available electronically to all members of the Dutch scientific associations involved in this CPG for commentary. Comments were collected and discussed and a second draft drawn up by the working group. Next, the Dutch patient/parent organization was asked to provide feedback, which led to a third draft. After reviewing and processing the comments, the final version of the CPGs was resubmitted to the scientific associations and approved.

The additional modules added between 2017 and 2019 followed the same methodology. After authorization, the CPG was published in the Dutch CPG database at www.richtlijnendatabase.nl.
Finally, the guidelines were translated into English by a grant from the European Reference Network/ERN-Cranio (www.ern-cranio.eu). Thus, the development of this CPG concerned a long process involving many steps. CPGs in the Netherlands are commonly checked every 5 years for the need to update. For this CPG, this will be done per module.

Reference

1 Guyatt, G.H.; Oxman, A.D.; Vist, G.; Kunz, R.; Brozek, J.; Alonso-Coello, P.; Montori, V.; Akl, E.A.; Djulbegovic, B.; Falck-Ytter, Y.; et al. GRADE guidelines: 4. Rating the quality of evidence—Study limitations (risk of bias). J. Clin. Epidemiol. 2011, 64. https://doi.org/10.1016/j.jclinepi.2010.07.017.

Supplementary Material S2

List of 60 references used for the conclusions of this guideline

(1) Augsornwan, D.; Surakunprapha, P.; Pattangtanang, P.; Pongpagatip, S.; Jenwitheesu, K.; Chowchuen, B.; Augsornwan, D.; Surakunprapha, P.; Pattangtanang, P.; Pongpagatip, S.; Jenwitheesuk, K.; Chowchuen, B. Comparison of Wound Dehiscence and Parent's Satisfaction between Spoon/Syringe Feeding and Breast/Bottle Feeding in Patients with Cleft Lip Repair. J. Med. Assoc. Thai. 2013, 96.

(2) Bessell, A.; Hooper, L.; Shaw, W. C.; Reilly, S.; Reid, J.; Glenny, A.-M. Feeding Interventions for Growth and Development in Infants with Cleft Lip, Cleft Palate or Cleft Lip and Palate. Cochrane Database Syst. Rev. 2011, No. 3. https://doi.org/10.1002/14651858.cd003315.pub3.

(3) Goyal, M.; Chopra, R.; Bansal, K.; Marwaha, M. Role of Obturators and Other Feeding Interventions in Patients with Cleft Lip and Palate: A Review. Eur. Arch. Paediatr. Dent. 2014, 15 (1). https://doi.org/10.1007/s40368-013-0101-0.

(4) Hughes, J.; Lindup, M.; Wright, S.; Naik, M.; Dhesi, R.; Howard, R.; Sommerlad, B.; Kangesu, L.; Sury, M. Does Nasogastric Feeding Reduce Distress after Cleft Palate Repair in Infants? Nurs. Child. Young People 2013, 25 (9). https://doi.org/10.7748/ncyp2013.11.25.9.26.e324.

(5) Ize-Iyamu, I. N.; Saheeb, B. D. Feeding Intervention in Cleft Lip and Palate Babies: A Practical Approach to Feeding Efficiency and Weight Gain. Int. J. Oral Maxillofac. Surg. 2011, 40 (9). https://doi.org/10.1016/j.ijom.2011.04.017.

(6) Jones, W. B. Weight Gain and Feeding in the Neonate with Cleft: A Three-Center Study. Cleft Palate J. 1988, 25 (4).

(7) Kim, E. K.; Lee, T. J.; Chae, S. W. Effect of Unrestricted Bottle-Feeding on Early Postoperative Course after Cleft Palate Repair. J. Craniofac. Surg. 2009, 20. https://doi.org/10.1097/SCS.0b013e3181b2d1d1.

(8) Turner, L.; Jacobsen, C.; Humenczuk, M.; Singhal, V. K.; Moore, D.; Bell, H. The Effects of Lactation Education and a Prosthetic Obturator Appliance on Feeding Efficiency in Infants with Cleft Lip and Palate. Cleft Palate-Craniofacial J. 2001, 38 (5). https://doi.org/10.1597/1545-1569(2001)038<0519:TEOLEA>2.0.CO;2.

(9) Bartzela, T.; Katsaros, C.; Shaw, W. C.; Rønning, E.; Rizell, S.; Bronkhorst, E.; Okada, T. O.; Pinheiro, F. H. D. S. L.; Dominguez-Gonzalez, S.; Hagberg, C.; Semb, G.; Kuijpers-Jagtman, A. M. A

Longitudinal Three-Center Study of Dental Arch Relationship in Patients with Bilateral Cleft Lip and Palate. Cleft Palate-Craniofacial J. 2010, 47 (2). https://doi.org/10.1597/08-249.1.
(10) Friede, H.; Enemark, H. Long-Term Evidence for Favorable Midfacial Growth after Delayed Hard Palate Repair in UCLP Patients. Cleft Palate-Craniofacial J. 2001, 38 (4). https://doi.org/10.1597/1545-1569(2001)038<0323:LTEFFM>2.0.CO;2.
(11) Fudalej, P.; Katsaros, C.; Bongaarts, C.; Dudkiewicz, Z.; Kuijpers-Jagtman, A. M. Dental Arch Relationship in Children with Complete Unilateral Cleft Lip and Palate Following One-Stage and Three-Stage Surgical Protocols. Clin. Oral Investig. 2011, 15 (4). https://doi.org/10.1007/s00784-010-0420-z.
(12) Grobbelaar, A. O.; Hudso, D. A.; Fernandes, D. B.; Lentin, R. Speech Results after Repair of the Cleft Soft Palate. Plast. Reconstr. Surg. 1995, 95 (7). https://doi.org/10.1097/00006534-199506000-00002.
(13) Gundlach, K. K. H.; Bardach, J.; Filippow, D.; Stahl-De Castrillon, F.; Lenz, J. H. Two-Stage Palatoplasty, Is It Still a Valuable Treatment Protocol for Patients with a Cleft of Lip, Alveolus, and Palate? J. Cranio-Maxillofacial Surg. 2013, 41 (1). https://doi.org/10.1016/j.jcms.2012.05.013.
(14) Kirschner, R. E.; Randall, P.; Wang, P.; Jawad, A. F.; Duran, M.; Huang, K.; Solot, C.; Cohen, M.; LaRossa, D. Cleft Palate Repair at 3 to 7 Months of Age. Plast. Reconstr. Surg. 2000, 105 (6). https://doi.org/10.1097/00006534-200005000-00032.
(15) Landheer, J. A.; Breugem, C. C.; Van Mink Der Molen, A. B. Fistula Incidence and Predictors of Fistula Occurrence after Cleft Palate Repair: Two-Stage Closure versus One-Stage Closure. Cleft Palate-Craniofacial J. 2010, 47 (6). https://doi.org/10.1597/09-069.
(16) Liao, Y. F.; Cole, T. J.; Mars, M. Hard Palate Repair Timing and Facial Growth in Unilateral Cleft Lip and Palate: A Longitudinal Study. Cleft Palate-Craniofacial J. 2006, 43 (5). https://doi.org/10.1597/05-119.
(17) Randag, A. C.; Dreise, M. M.; Ruettermann, M. Surgical Impact and Speech Outcome at 2.5 Years after One- or Two-Stage Cleft Palate Closure. Int. J. Pediatr. Otorhinolaryngol. 2014. https://doi.org/10.1016/j.ijporl.2014.08.021.
(18) Richard, B.; Russell, J.; McMahon, S.; Pigott, R. Results of Randomized Controlled Trial of Soft Palate First versus Hard Palate First Repair in Unilateral Complete Cleft Lip and Palate. Cleft Palate-Craniofacial J. 2006, 43 (3). https://doi.org/10.1597/05-065.1.
(19) Rohrich, R. J.; Rowsell, A. R.; Johns, D. F.; Drury, M. A.; Grieg, G.; Watson, D. J.; Godfrey, A. M.; Poole, M. D. Timing of Hard Palatal Closure: A Critical Long-Term Analysis. Plast. Reconstr. Surg. 1996. https://doi.org/10.1097/00006534-199608000-00005.
(20) Wada, T.; Tachimura, T.; Satoh, K.; Hara, H.; Hatano, M.; Sayan, N. B.; Tatsuta, U. Maxillary Growth after Two-Stage Palatal Closure in Complete (Unilateral and Bilateral) Clefts of the Lip and Palate from Infancy until 10 Years of Age. J. Osaka Univ. Dent. Sch. 1990.
(21) Yang, I. Y.; Liao, Y. F. The Effect of 1-Stage versus 2-Stage Palate Repair on Facial Growth in Patients with Cleft Lip and Palate: A Review. Int. J. Oral Maxillofac. Surg. 2010, 39 (10). https://doi.org/10.1016/j.ijom.2010.04.053.
(22) Ysunza, A.; Pamplona, M. C.; Mendoza, M.; García-Velasco, M.; Aguilar, M. P.; Guerrero, M. E. Speech Outcome and Maxillary Growth in Patients with Unilateral Complete Cleft Lip/Palate Operated on at 6 versus 12 Months of Age. Plast. Reconstr. Surg. 1998, 112 (3). https://doi.org/10.1097/00006534-199809010-00009.

(23) Zemann, W.; Kärcher, H.; Drevenšek, M.; Koželj, V. Sagittal Maxillary Growth in Children with Unilateral Cleft of the Lip, Alveolus and Palate at the Age of 10 Years: An Intercentre Comparison. J. Cranio-Maxillofacial Surg. 2011. https://doi.org/10.1016/j.jcms.2010.10.025.
(24) Williams, W. N.; Seagle, M. B.; Pegoraro-Krook, M. I.; Souza, T. V.; Garla, L.; Silva, M. L.; MacHado Neto, J. S.; Dutka, J. C. R.; Nackashi, J.; Boggs, S.; Shuster, J.; Moorhead, J.; Wharton, W.; Graciano, M. I. G.; Pimentel, M. C.; Feniman, M.; Piazentin-Penna, S. H. A.; Kemker, J.; Zimmermann, M. C.; Bento-Gonçalvez, C.; Borgo, H.; Marques, I. L.; Martinelli, A. P. M. C.; Jorge, J. C.; Antonelli, P.; Neves, J. F. A.; Whitaker, M. E. Prospective Clinical Trial Comparing Outcome Measures between Furlow and von Langenbeck Palatoplasties for UCLP. Ann. Plast. Surg. 2011, 66 (2). https://doi.org/10.1097/SAP.0b013e3181d60763.
(25) Henkel, K. O.; Dieckmann, A.; Dieckmann, O.; Lenz, J. H.; Gundlach, K. K. H. Veloplasty Using the Wave-Line Technique Versus Classic Intravelar Veloplasty. Cleft Palate-Craniofacial J. 2004, 41 (1). https://doi.org/10.1597/02-011.
(26) Abdel-Aziz, M.; Ghandour, H. Comparative Study between V-Y Pushback Technique and Furlow Technique in Cleft Soft Palate Repair. Eur. J. Plast. Surg. 2011, 34 (1). https://doi.org/10.1007/s00238-010-0443-3.
(27) Hassan, M. E.; Askar, S. Does Palatal Muscle Reconstruction Affect the Functional Outcome of Cleft Palate Surgery? Plast. Reconstr. Surg. 2007, 119 (6). https://doi.org/10.1097/01.prs.0000259185.29517.79.
(28) Witt, P. D.; Cohen, D. T.; Muntz, H. R.; Grames, L. M.; Pilgram, T. K.; Marsh, J. L. Long-Term Stability of Postpalatoplasty Perceptual Speech Ratings: A Prospective Study. Ann. Plast. Surg. 1999, 43 (3). https://doi.org/10.1097/00000637-199909000-00004.
(29) McWilliams, B. J.; Randall, P.; LaRossa, D.; Cohen, S.; Yu, J.; Cohen, M.; Solot, C. Speech Characteristics Associated with the Furlow Palatoplasty as Compared with Other Surgical Techniques. Plast. Reconstr. Surg. 1996, 98 (4). https://doi.org/10.1097/00006534-199609001-00003.
(30) Carroll, D. J.; Padgitt, N. R.; Liu, M.; Lander, T. A.; Tibesar, R. J.; Sidman, J. D. The Effect of Cleft Palate Repair Technique on Hearing Outcomes in Children. Int. J. Pediatr. Otorhinolaryngol. 2013. https://doi.org/10.1016/j.ijporl.2013.06.021.
(31) Spauwen, P. H. M.; Goorhuis-Brouwer, S. M.; Schutte, H. K. Cleft Palate Repair: Furlow versus von Langenbeck. J. Cranio-Maxillofacial Surg. 1992. https://doi.org/10.1016/S1010-5182(05)80190-8.
(32) Reddy, S. G.; Reddy, R. R.; Bronkhorst, E. M.; Prasad, R.; Kuijpers Jagtman, A. M.; Bergé, S. Comparison of Three Incisions to Repair Complete Unilateral Cleft Lip. Plast. Reconstr. Surg. 2010. https://doi.org/10.1097/PRS.0b013e3181d45143.
(33) Chowdri, N. A.; Darzi, M. A.; Ashraf, M. M. A Comparative Study of Surgical Results with Rotation-Advancement and Triangular Flap Techniques in Unilateral Cleft Lip. Br. J. Plast. Surg. 1990. https://doi.org/10.1016/0007-1226(90)90119-K.
(34) De Silva Amaratunga, N. A. Combining Millard's and Cronin's Methods of Unilateral Cleft Lip Repair - A Comparative Study. Asian J. Oral Maxillofac. Surg. 2004. https://doi.org/10.1016/S0915-6992(04)80001-3.
(35) Halli, R.; Joshi, A.; Kini, Y.; Kharkar, V.; Hebbale, M. Retrospective Analysis of Sutureless Skin Closure in Cleft Lip Repair. J. Craniofac. Surg. 2012. https://doi.org/10.1097/scs.0b013e318241db01.

(36) Kuo, C. L.; Tsao, Y. H.; Cheng, H. M.; Lien, C. F.; Hsu, C. H.; Huang, C. Y.; Shiao, A. S. Grommets for Otitis Media with Effusion in Children with Cleft Palate: A Systematic Review. Pediatrics 2014, 134 (5). https://doi.org/10.1542/peds.2014-0323.

(37) Ponduri, S.; Bradley, R.; Ellis, P. E.; Brookes, S. T.; Sandy, J. R.; Ness, A. R. The Management of Otitis Media with Early Routine Insertion of Grommets in Children with Cleft Palate-a Systematic Review. Cleft Palate-Craniofacial J. 2009, 46 (1). https://doi.org/10.1597/07-219.1.

(38) Ysunza, A.; Pamplona, M. C.; Molina, F.; Drucker, M.; Felemovicius, J.; Ramírez, E.; Patiño, C. Surgery for Speech in Cleft Palate Patients. Int. J. Pediatr. Otorhinolaryngol. 2004, 68 (12). https://doi.org/10.1016/j.ijporl.2004.06.010.

(39) Åbyholm, F.; D'Antonio, L.; Ward, S. L. D.; Kjøll, L.; Saeed, M.; Shaw, W. C.; Sloan, G.; Whitby, D.; Worthington, H.; Wyatt, R. Pharyngeal Flap and Sphincterplasty for Velopharyngeal Insufficiency Have Equal Outcome at 1 Year Postoperatively: Results of a Randomized Trial. Cleft Palate-Craniofacial J. 2005, 42 (5). https://doi.org/10.1597/03-148.1.

(40) Dempf, R.; Teltzrow, T.; Kramer, F. J.; Hausamen, J. E. Alveolar Bone Grafting in Patients with Complete Clefts: A Comparative Study between Secondary and Tertiary Bone Grafting. Cleft Palate-Craniofacial J. 2002, 39 (1). https://doi.org/10.1597/1545-1569(2002)039<0018:ABGIPW>2.0.CO;2.

(41) Freihofer, H. P. M.; Borstlap, W. A.; Kuijpers-Jagtman, A. M.; Voorsmit, R. A. C. A.; van Damme, P. A.; Heidbüchel, K. L. W. M.; Borstlap-Engels, V. M. F. Timing and Transplant Materials for Closure of Alveolar Clefts. A Clinical Comparison of 296 Cases. J. Cranio-Maxillofacial Surg. 1993, 21 (3). https://doi.org/10.1016/S1010-5182(05)80102-7.

(42) Jia, Y. L.; Fu, M. K.; Ma, L. Long-Term Outcome of Secondary Alveolar Bone Grafting in Patients with Various Types of Cleft. Br. J. Oral Maxillofac. Surg. 2006, 44 (4). https://doi.org/10.1016/j.bjoms.2005.07.003.

(43) Nishihara, K.; Nozoe, E.; Maeda, A.; Hirahara, N.; Okawachi, T.; Miyawaki, S.; Nakamura, N. Original Article Outcome Following Secondary Autogenous Bone Grafting before and after Canine Eruption in Patients with Unilateral Cleft Lip and Palate. Cleft Palate-Craniofacial J. 2014, 51 (2). https://doi.org/10.1597/11-298.

(44) Miller, L. L.; Kauffmann, D.; St. John, D.; Wang, D.; Grant, J. H.; Waite, P. D. Retrospective Review of 99 Patients With Secondary Alveolar Cleft Repair. J. Oral Maxillofac. Surg. 2010, 68 (6). https://doi.org/10.1016/j.joms.2009.09.106.

(45) Rawashdeh, M. A.; Al Nimri, K. S. Outcome of Secondary Alveolar Bone Grafting before and after Eruption of the Canine in Jordanian Patients with Cleft Lip and Palate. J. Craniofac. Surg. 2007, 18 (6). https://doi.org/10.1097/scs.0b013e31814e059b.

(46) Sindet-Pedersen, S. Comparative Study of Secondary and Late Secondary Bone-Grafting in Patients with Residual Cleft Defects. Short-Term Evaluation. Int. J. Oral Surg. 1985, 14 (5). https://doi.org/10.1016/S0300-9785(85)80071-5.

(47) Trindade-Suedam, I. K.; Da Silva Filho, O. G.; Carvalho, R. M.; De Souza Faco, R. A.; Calvo, A. M.; Ozawa, T. O.; Trindade, A. S.; Kiemle Trindade, I. E. Timing of Alveolar Bone Grafting Determines Different Outcomes in Patients with Unilateral Cleft Palate. J. Craniofac. Surg. 2012, 23 (5). https://doi.org/10.1097/SCS.0b013e3182519ab5.

(48) Alonso, N.; Tanikawa, D. Y. S.; Freitas, R. D. S.; Canan, Lady; Ozawa, T. O.; Rocha, D. L. Evaluation of Maxillary Alveolar Reconstruction Using a Resorbable Collagen Sponge with

Recombinant Human Bone Morphogenetic Protein-2 in Cleft Lip and Palate Patients. Tissue Eng. - Part C Methods 2010, 16 (5). https://doi.org/10.1089/ten.tec.2009.0824.

(49) Canan, L. W.; Da Silva Freitas, R.; Alonso, N.; Tanikawa, D. Y. S.; Rocha, D. L.; Coelho, J. C. U. Human Bone Morphogenetic Protein-2 Use for Maxillary Reconstruction in Cleft Lip and Palate Patients. J. Craniofac. Surg. 2012, 23 (6). https://doi.org/10.1097/SCS.0b013e31825c75ba.

(50) Enemark, H.; Jensen, J.; Bosch, C. Mandibular Bone Graft Material for Reconstruction of Alveolar Cleft Defects: Long-Term Results. Cleft Palate-Craniofacial J. 2001, 38 (2). https://doi.org/10.1597/1545-1569(2001)038<0155:MBGMFR>2.0.CO;2.

(51) Thuaksuban, N.; Nuntanaranont, T.; Pripatnanont, P. A Comparison of Autogenous Bone Graft Combined with Deproteinized Bovine Bone and Autogenous Bone Graft Alone for Treatment of Alveolar Cleft. Int. J. Oral Maxillofac. Surg. 2010, 39 (12). https://doi.org/10.1016/j.ijom.2010.07.008.

(52) Liang, Z.; Yao, J.; Chen, P. K. T.; Zheng, C.; Yang, J. Effect of Presurgical Nasoalveolar Molding on Nasal Symmetry in Unilateral Complete Cleft Lip/Palate Patients after Primary Cheiloplasty without Concomitant Nasal Cartilage Dissection: Early Childhood Evaluation. Cleft Palate-Craniofacial J. 2018, 55 (7). https://doi.org/10.1597/14-296.

(53) Shetty, V.; Agrawal, R. K.; Sailer, H. F. Long-Term Effect of Presurgical Nasoalveolar Molding on Growth of Maxillary Arch in Unilateral Cleft Lip and Palate: Randomized Controlled Trial. Int. J. Oral Maxillofac. Surg. 2017, 46 (8). https://doi.org/10.1016/j.ijom.2017.03.006.

(54) Borzabadi-Farahani, A.; Lane, C. J.; Yen, S. L. K. Late Maxillary Protraction in Patients with Unilateral Cleft Lip and Palate: A Retrospective Study. Cleft Palate-Craniofacial J. 2014, 51 (1). https://doi.org/10.1597/12-099.

(55) Susami, T.; Okayasu, M.; Inokuchi, T.; Ohkubo, K.; Uchino, N.; Uwatoko, K.; Takahashi-Ichikawa, N.; Nagahama, K.; Takato, T. Maxillary Protraction in Patients with Cleft Lip and Palate in Mixed Dentition: Cephalometric Evaluation after Completion of Growth. Cleft Palate-Craniofacial J. 2014, 51 (5). https://doi.org/10.1597/12-032.

(56) Marcusson, A.; Paulin, G. Changes in Occlusion and Maxillary Dental Arch Dimensions in Adults with Treated Unilateral Complete Cleft Lip and Palate: A Follow-up Study. Eur. J. Orthod. 2004, 26 (4). https://doi.org/10.1093/ejo/26.4.385.

(57) Kloukos, D.; Fudalej, P.; Sequeira-Byron, P.; Katsaros, C. Maxillary Distraction Osteogenesis versus Orthognathic Surgery for Cleft Lip and Palate Patients. Cochrane Database Syst. Rev. 2016, 9. https://doi.org/10.1002/14651858.CD010403.pub2.

(58) Norman, A.; Persson, M.; Stock, N.; Rumsey, N.; Sandy, J.; Waylen, A.; Edwards, Z.; Hammond, V.; Partridge, L.; Ness, A. The Effectiveness of Psychosocial Intervention for Individuals with Cleft Lip and/or Palate. Cleft Palate-Craniofacial J. 2015, 52 (3). https://doi.org/10.1597/13-276.

(59) Hunt, O.; Burden, D.; Hepper, P.; Johnston, C. The Psychosocial Effects of Cleft Lip and Palate: A Systematic Review. Eur. J. Orthod. 2005, 27 (3). https://doi.org/10.1093/ejo/cji004.

(60) Nolet, P. J. P. M.; Katsaros, C.; Van't Hof, M. A.; Kuijpers-Jagtman, A. M. Treatment Outcome in Unilateral Cleft Lip and Palate Evaluated with the GOSLON Yardstick: A Meta-Analysis of 1236 Patients. Plast. Reconstr. Surg. 2005, 116 (5). https://doi.org/10.1097/01.prs.0000181652.84855.a3.

Supplementary Material S3

Current gaps in knowledge

During the development of this CPG, we systematically searched the literature to find answers to the clinical questions. Some (or parts) of the clinical questions were answered by the results of these searches, but many were not answered. Using an evidence-based method of guideline development, important gaps in knowledge in the field of cleft care were identified due to a lack of power of most studies and/or many studies grouping different cleft subphenotypes as one entity. Therefore, the guideline working group holds the opinion that further study is needed and has listed the most important knowledge gaps per chapter below.

Genetic testing

- Absence of an optimum genetic testing strategy.

Feeding

- Uncertainty exists about the best method of administering food to babies with cleft lip, alveolus, and/or palate.

Lip and palate closure

- Uncertainty exists about the best timing for lip closure and palate closure.
- Uncertainty exists about the best surgical technique for lip and palate closure.

Hearing problems

- Uncertainty exists regarding the effects of early ventilation tube insertion in children with a cleft lip, alveolus, and palate.

Hypernasality

- Uncertainty exists regarding the best diagnostic strategy for velopharyngeal dysfunction (VPD) in children with a cleft deformity involving the palate.
- Uncertainty exists regarding the best surgical technique for treating VPD in children with a cleft involving the palate.

Bone grafting procedures

- Uncertainty exists about the best timing for bone grafting in the alveolar cleft.
- Uncertainty exists about using specific bone transplant materials for bone grafting in the alveolar cleft.

Orthodontic treatment

- Uncertainty exists about the timing and effect of maxillary protraction for midfacial deficiency due to an absence of long-term follow-up studies until facial growth is complete.
- Uncertainty exists about the most effective long-term retention strategy in children with cleft lip, alveolus, and palate for retaining teeth positions and the width of the maxillary dental arch.

Nasoalveolar molding

- Whether naso-alveolar molding (NAM) has a lasting positive effect on the shape and function of the nose in children with a complete unilateral or bilateral cleft has not been sufficiently investigated.
- Whether NAM promotes the psychosocial well-being of parents or facilitates lip surgery has not been proven.
- Cost effectiveness must also be investigated.

Rhinoplasty

- Uncertainty exists about the rhinoplasty treatment strategy that achieves the best outcome in children with cleft lip, alveolus, and palate.

Psychosocial guidance

- Uncertainty exists about the best timing for psychosocial guidance during the child's development.

Osteotomy versus distraction osteogenesis

- The effect of Le Fort I osteotomy versus distraction osteogenesis on several outcome variables, such as speech, bony and dental relapse, revision surgery, masticatory function, and post-operative relation between the bone and soft tissues, has not been sufficiently studied in cleft patients.
